# Alterations of the Enteric Virome in Vogt-Koyanagi-Harada Disease

**DOI:** 10.1167/iovs.66.6.15

**Published:** 2025-06-04

**Authors:** Mingzhu Liu, Jiawei Geng, Siyan Jin, Ping Hu, Xia Wang, Xiaoli Liu

**Affiliations:** 1 Ophthalmologic Center of the Second Hospital, Jilin University, Changchun, People's Republic of China

**Keywords:** *Pseudomonas* phage, enteric virome, Vogt-Koyanagi-Harada disease (VKH), Shotgun metagenomic sequencing

## Abstract

**Purpose:**

This study aims to explore the enteric virome characteristics of Vogt Koyanagi Harada (VKH) disease and its potential role in this disease.

**Methods:**

Shotgun metagenomic sequencing was used to detect the enteric virome and 16S rRNA to detect the bacteriome in new-onset, untreated patients with VKH (*n* = 25) and age- and sex-matched healthy controls without autoimmune diseases (*n* = 25).

**Results:**

Patients with VKH exhibited different enteric viral communities from healthy controls, characterized by decreased richness of core viral communities (present in > 80% of samples) and increased richness of common viral communities (present in 50%–80% of samples). Notably, within the core virus community, bacteriophage richness was markedly reduced, whereas eukaryotic virus richness significantly increased in patients with VKH. The case-control analysis identified 42 differentially abundant viruses, including a decrease in crAss-like phages, the eukaryotic virus *Moumouvirus_moumou*, and an enrichment of the *Chlamydiamicrovirus_CPG1*. Most of the differential phages predominantly targeted bacteria from the phyla *Pseudomonadota* and *Firmicutes*. The gut virome-bacteria community correlation analysis revealed a shift in the interactions between the core viruses and bacterial communities. Additionally, *Wroclawvirus PA5oct* (a *Pseudomonas* phage) correlated with leukotrichia, a clinically relevant symptom of VKH (*P* = 0.042). The impact of multiple *Pseudomonas* phages on the host folate biosynthesis was significantly enhanced in patients with VKH. Moreover, the protein (Earp_361-372_) encoded by VKH-enriched *Pseudomonas* was identified to share homology with the melanin antigen gp100_44-59_.

**Conclusions:**

The gut virome of patients with VKH differs significantly from healthy controls, suggesting its disturbance may contribute to gut microbiome imbalance and VKH development.

Vogt-Koyanagi-Harada (VKH) disease is a T-cell-mediated autoimmune disorder targeting melanocyte-associated antigens, with primary target organs including the eyes, ears, meninges, and skin.^1^ This condition accounts for 6% to 8% of uveitis cases in Asia, a proportion higher than in other regions.^2^ The exact etiology and pathogenesis of VKH disease remain unclear, but most studies suggest that it may be related to genetics,^3^ immunity,^1^ and various environmental factors including intestinal microorganisms.^4^^,^^5^ Previous studies found that patients with VKH exhibited specific intestinal flora and metabolite alterations.^4^^–^^7^ Other research has also demonstrated that fecal microbiota from patients with VKH can exacerbate the disease severity of experimental autoimmune uveitis (EAU) mice through fecal transplantation experiments.^5^ However, the gut microbiota not only includes bacterial components but also contains important viral components. The role of the gut virome in the pathogenesis of VKH disease has yet to be explored.

Importantly, studies have proposed an association between virus infections and the pathogenesis of VKH. Bassili et al. have isolated Epstein-Barr virus (EBV) DNA from the vitreous fluids of patients with VKH by PCR.^8^ Moreover, T cells from patients with VKH exhibited cross-reactivity between tyrosinase peptides and highly homologous cytomegalovirus (CMV) antigen.^9^^,^^10^ These indicate that virus infections may act as a potential trigger, leading to the development of VKH.^10^ However, it is unclear whether there is viral dysbiosis in the gut of patients with VKH, and whether there is an interaction between changes in the virome and VKH pathogenesis remains unknown. Indeed, gut virome disorders have been observed in various autoimmune or inflammatory diseases, including systemic lupus erythematosus (SLE), rheumatoid arthritis (RA), inflammatory bowel disease (IBD), and type 2 diabetes mellitus (T2D).^11^^–^^14^ For example, in patients with IBD, bacteriophage changes have been focused on, and a notable expansion of *Caudovirales* phage was found in both fecal and mucosal virome.^13^^,^^15^ Fecal or mucosal virus-like particle (VLP) transplantation from patients with IBD can exacerbate intestinal inflammation by remodeling gut bacteriome-virome ecology.^16^^,^^17^ In this study, we performed shotgun metagenomic sequencing on new-onset, untreated patients with VKH and healthy controls to compare their gut viral communities, and analyzed the association between gut bacteriome and virome. We have, for the first time, depicted the overall characteristics of the gut virome in VKH disease, and explored the potential mechanisms of the gut virome in the pathogenesis of VKH.

## Materials and Methods

### Participants, and Fecal Sample Collection

New-onset, untreated patients with active VKH (the VKH group, *n* = 25) and age- and sex-matched healthy controls (the N group, *n* = 25) who visited the Department of Ophthalmology, the Second Hospital of Jilin University were included. The diagnosis of VKH disease was based on the revised criteria of the International Committee,^18^ combined with the modified criteria by the Chinese team.^19^ Patients with new-onset and active VKH presented with characteristic early phase ocular manifestations, such as diffuse choroiditis and exudative retinal detachment.^18^^,^^19^ The inclusion or exclusion criteria for the N group and the new-onset, untreated, and active VKH group were consistent with previously described methods.^4^ In brief, all subjects had not received any medications, including antibiotics or supplements, within 6 months before the collection of fecal samples. Individuals with infectious diseases or immune diseases, such as IBD and RA, were excluded. The virome cohort included 10 patients with VKH (mean age = 51.8 ± 10.68, male/female = 0.67/1), and 10 healthy controls (mean age = 46.6 ± 15.14, male/female = 0.67/1). The bacteriome cohort included 15 patients with VKH (mean age = 48.13 ± 13.05, male/female = 0.67/1), and 15 healthy controls (mean age = 41.93 ± 12.00, male/female = 1.14/1). There was no significant difference in age and sex between the VKH and the N groups in either cohort. All patients with VKH had at least one extraocular manifestation, including poliosis (16/23), alopecia (9/23), tinnitus (5/23), hearing loss (10/23), meningitis (11/23), and vitiligo (2/23). All subjects who participated in this study provided written informed consent. All procedures followed the principles of the Declaration of Helsinki and were approved by the Medical Ethics Committee of the Second Hospital of Jilin University (2025-061). Fecal samples were collected into sterile fecal tubes after defecation by all participants, and the obtained samples were stored at –80°C until use.

### Bacterial and Viral DNA Extraction and Sequencing

Gut bacteriome was sequenced by 16S rRNA. Bacterial DNA extraction, library preparation and sequencing, and bioinformatics analysis were conducted, as described in Ref. 4. In brief, bacteriome DNA was extracted using Cetyltrimethylammonium bromide. The size and quantity of the amplicon library were measured using the Agilent 2100 bioanalyzer (Agilent, USA) and the Illumina Library Quantification Kit (Kapa Biosciences, Woburn, MA, USA), respectively. Bacteriome libraries were then sequenced on the Illumina NovaSeq platform.

Virome was analyzed by Shotgun metagenomic sequencing. Fecal samples from each subject were frozen in liquid nitrogen and ground. PBS buffer (equal volume to the sample) was added to the grinding tube, and the mixture was vortexed. The homogenized sample was then centrifuged, and 500 µL of the supernatant was filtered through a 0.45 µm filter (Millipore) to remove particles of eukaryotic and bacterial cell sizes. According to the manufacturer’s instructions, viral DNA was extracted using the DNeasy PowerSoil Pro Kit Manual Virus Extraction Kit. A metagenomic high-throughput sequencing library was then constructed using the TruSeq Nano DNA LT Library Prep Kit (Illumina). Shotgun metagenome sequencing was performed on the Illumina NovaSeq platform of Shanghai Personalbio Technology Co., Ltd. The raw data of metagenomic sequencing data has been deposited in the National Center for Biotechnology Information website under BioProject number PRJNA1225217 at https://www.ncbi.nlm.nih.gov/bioproject/PRJNA1225217.

### Bioinformatics Analyses of Virome

Raw reads were preprocessed to obtain high-quality reads for subsequent analyses. First, sequencing adapters were removed from sequencing reads using Cutadapt (version 1.17). Second, low-quality reads were trimmed using a sliding-window algorithm in Fastp (version 0.20.0) and used to BLASTN with NCBI virus reference genome database to identify the candidate viral sequences (e-value = < 1e-5). Contigs were assembled from reads of each sample by using metaSPAdes (version 3.13.0) with default parameters.^20^ Non-redundant contigs were generated by using MMseqs in “linclust” mode, setting the sequence identification threshold to 0.98 and residue coverage of the shorter contig to 95%.

Non-redundant contigs for viral contig prediction: (1) viral sequences were identified using default parameter settings in VIBRANT,^21^ (2) contigs with scores ≥ 0.9 in VirSorter2 were classified as viral contigs,^22^ and (3) contigs with viral gene content exceeding microbial gene content were confirmed as viral by CheckV.^23^ Gene prediction for viral contigs was performed using TransGeneScan.^24^ The bacterial host of the target phage was identified by querying the Virus-Host DB (https://www.genome.jp/virushostdb/note.html).^25^

Species annotation of the protein sequences of genes was performed using MMseqs2 in “taxonomy” mode by aligning against the NCBI-NT database. The high-quality reads from each sample were mapped onto the predicted gene sequences using minimap2,^26^ and the transcripts per kilobase per million mapped reads (TPM) was calculated to normalize the abundance of virome genes. MMseqs2 was used to perform homology comparison of protein sequences with the Kyoto Encyclopedia of Genes and Genomes (KEGG) database to obtain functional gene annotations.

### Melanocyte Antigen Homology

To investigate whether proteins encoded by gut microbiota share sequence homology with melanocyte antigens, tyrosinase_450–462_ (SYLQDSDPDSFQD) and gp100_44-59_ (WNRQLYPEWTEAQRLD) were input as query sequences.^10^ Homology analysis was performed using the BLASTP program against the NCBI protein database. The resulting hits were further examined to determine whether any homologous sequences originated from the virus or bacteria were identified in this study.

### Statistical Analyses

The statistical analyses and visualization processes involved in this study were completed by QIIME2 (https://qiime2.org), R software (version 4.1.1), and GraphPad Prism (version 9.5.1). The α diversity and β diversity were calculated using the vegan R software package. QIIME was used to visualize the Principal coordinates analysis (PCoA) analysis. Permutational multivariate analysis of variance (PERMANOVA) analysis was performed using the Adonis function. The Wilcox. test function of the stats R package was used to compare differential viruses between patients with VKH and healthy controls (*P* < 0.05, |logFC| ≥ 1). Spearman’s correlation analysis was performed using the cor function. GraphPad Prism was used to calculate the area under the curve (AUC) to evaluate the classification model performance of the *Pseudomonas* phage.

## Results

### Diversity of Enteric Virome in Patients With VKH 

**Figure 1.**
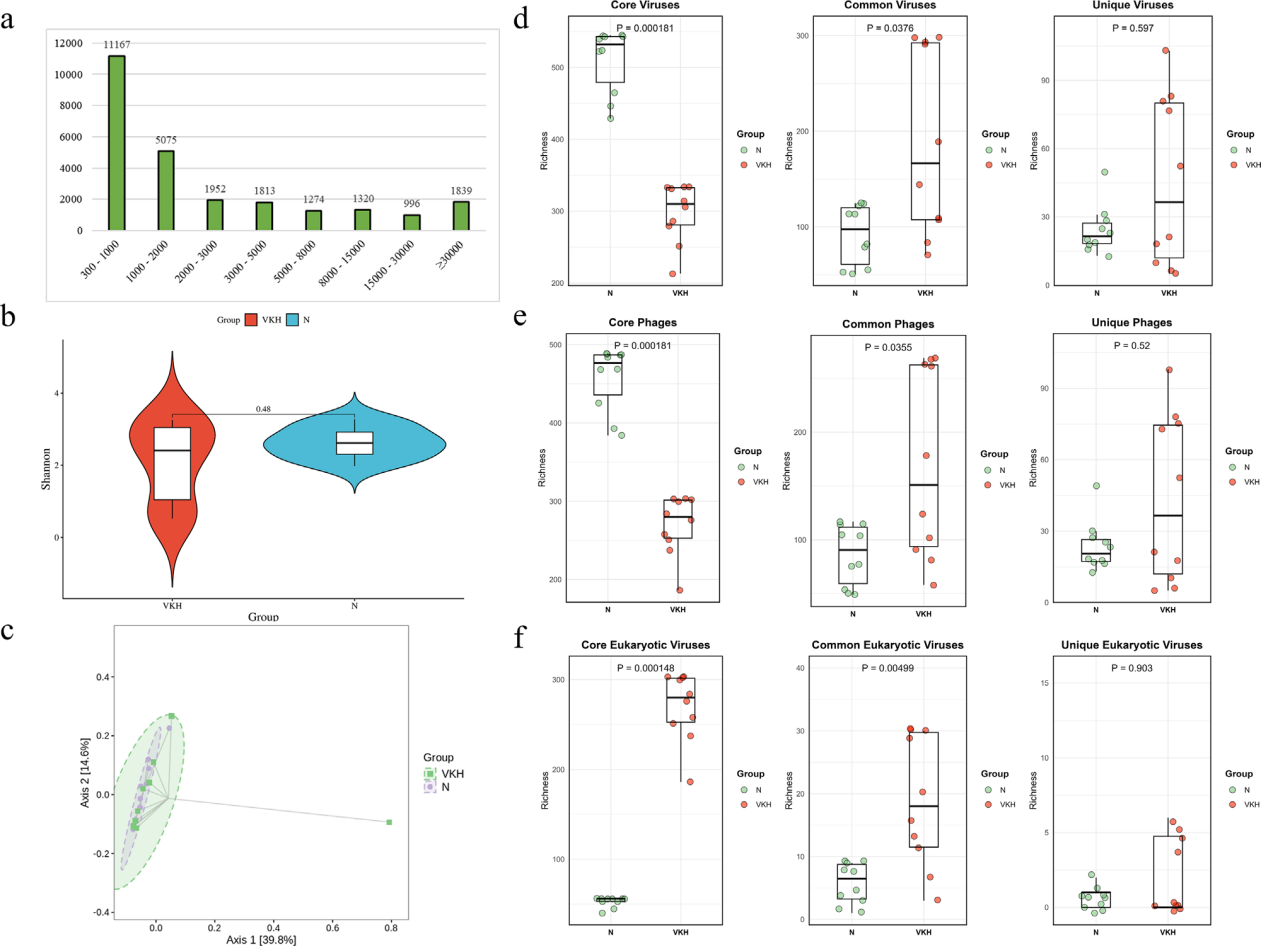
Viral diversity in the VKH and the N groups. (**a**) Distribution of contig lengths in the enteric virome. (**b**) Shannon index analysis of the whole gut virus communities. (**c**) PCoA plotting of the whole gut virus communities based on the Bray-Curtis distance. (**d**) Richness analysis of three virus communities (core, common, and unique) between the N and the VKH groups. (**e**) Richness analysis of three phage communities (core, common, and unique) between the N and the VKH groups. (**f**) Richness analysis of three eukaryotic virus communities (core, common, and unique) between the N and the VKH groups.

Altogether, 1.508 billion raw sequencing reads were generated through shotgun metagenomic sequencing. After de novo assembly and strict filtering, a total of 38,371 viral clusters were identified, with the length distribution mainly concentrated between 300 and 1000 bp (Fig. 1a).

We first evaluated the diversity in the gut virome between the VKH and the N groups. The α diversity (Shannon index) was not significantly altered in the VKH group (Fig. 1b). PCoA (based on Bray-Curtis Distance) indicated no significant difference in viral composition between the two groups (Adonis, *P* = 0.718; Fig. 1c). We also analyzed the effects of age and gender on the α diversity of the gut virome. The results showed no significant correlation between age or gender and the four indices (Chao1, Shannon, Simpson, and Ace;Supplementary Fig. S1).

Using previously reported criteria,^27^ the overall enteric virome was divided into three communities: ① core virome community: virus species present in over 80% of the samples; ② common virome community: those in 50% to 80%; and ③ unique virome community: those in < 50%. The α diversity indices of the three viral communities were separately compared between the two groups. In the VKH group, the richness of core viruses decreased notably, whereas the richness of common viruses increased significantly, and the richness of unique viruses showed no significant change (Fig. 1d). Shannon index of the three viral communities did not differ significantly between the two groups (Supplementary Fig. S2). The similar results were observed in the comparison of bacteriophage between the VKH and the N groups (Fig. 1e). Conversely, the richness of the core eukaryotic virus community was notably increased in the VKH group (Fig. 1f).

### Composition and Abundance of Enteric Virome in Patients With VKH 

**Figure 2.**
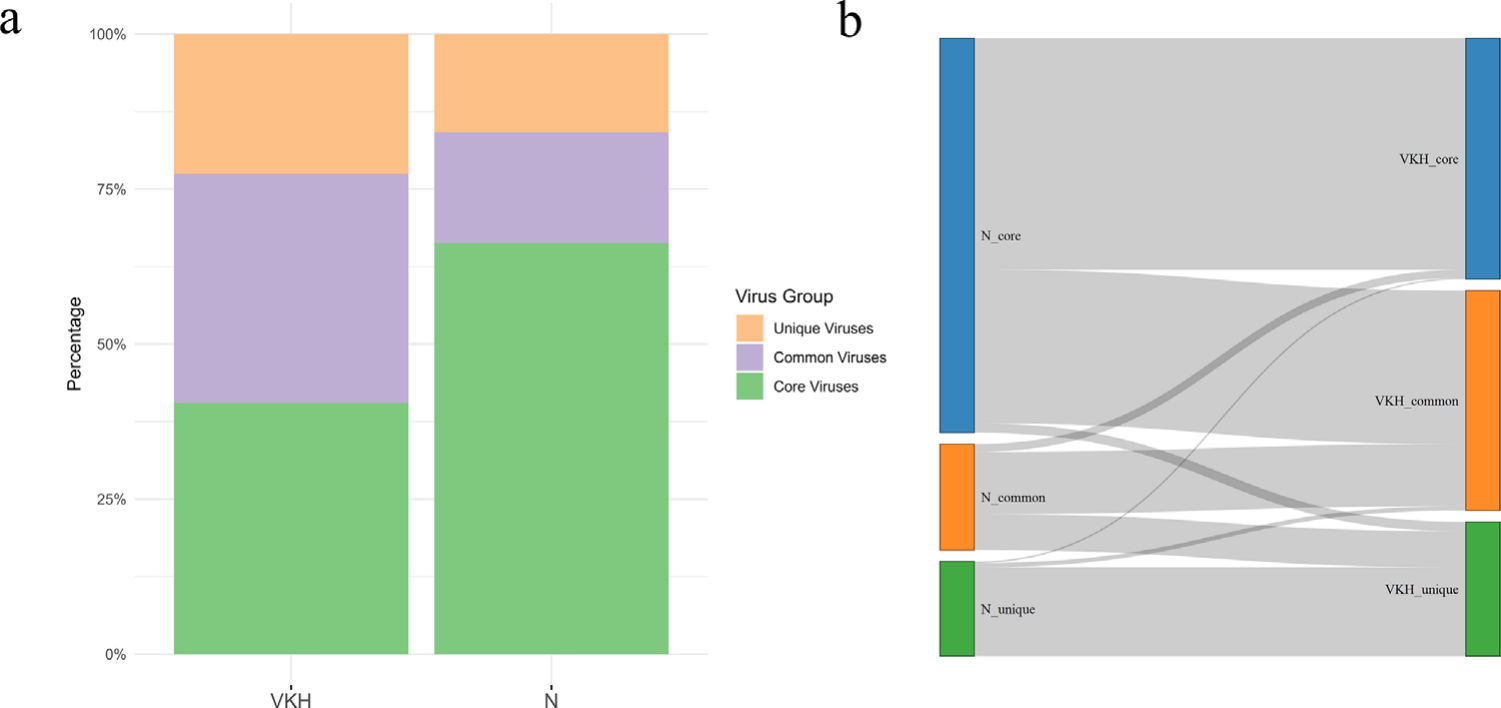
Composition of enteric virome in the VKH and the N groups. (**a**) The proportion of core, common, and unique viruses in the whole gut virus communities. (**b**) The Sankey diagram showing the transition of three virus communities (core, common, and unique) between the N and the VKH groups.

The composition of the three viral communities (core, common, and unique communities) in the N group and the VKH group was compared (Fig. 2a). It was found that 213 of 547 core viruses in the N group transitioned to common viruses in patients with VKH, whereas 50 of 147 common viruses in the N group became unique viruses in patients with VKH (Fig. 2b). The Sankey diagram indicated that in patients with VKH, the dominance of the core virome gradually weakened, with a shift toward common viruses.

**Figure 3.**
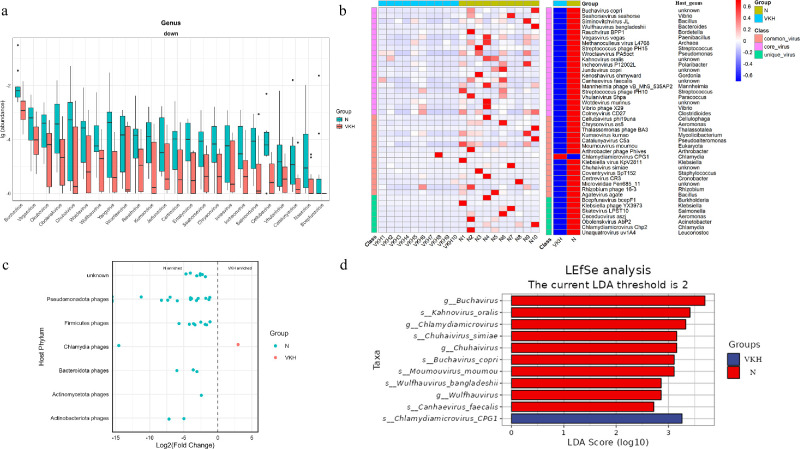
Differential viral taxa between the VKH and the N groups. Differentially abundant viruses at the genus level (**a**), and species level (**b**) between the VKH and the N groups (Wilcoxon test, only those differential taxa with *P* < 0.05 and |logFC| ≥ 1 are shown). (**c**) Differentially abundant phages in VKH for which the bacterial hosts could be predicted were displayed at the phylum level. (**d**) Differentially abundant viruses by LEfSe analysis.

Next, we identified viruses differentially expressed in the VKH and the N groups. At the genus level, 24 viruses were significantly deleted in patients with VKH (Fig. 3a). At the species level, patients with VKH had 42 differential viruses (41 downregulated and 1 upregulated; Fig. 3b). These differential species were mainly members of the families *Suoliviridae*, *Steigviridae*, *Straboviridae*, and *Microviridae*. Host assignment analysis revealed that differential phages targeting *Pseudomonadota* and *Firmicutes* were the most common (Fig. 3c), which was consistent with the overall enteric phage composition in patients with VKH. Additionally, the differentially expressed viruses consisted of 20 core viruses, 15 common viruses, and 7 unique viruses. The above results indicate that the gut virome composition in patients with VKH is significantly dysregulated at the taxonomic composition.

Linear Discriminant Analysis Effect Size (LEfSe) analysis was further performed to evaluate the contribution of viruses to the differences between the two groups (Fig. 3d). Only *s_Chlamydiamicrovirus_CPG1*, a phage that hosts Chlamydia, was significantly enriched in the VKH group. Meanwhile, 10 taxa were significantly enriched in the N group, and *g_ Buchavirus* contributed the most to the difference between the 2 groups. The differential viruses decreased in the VKH group were mainly divided into two categories: Nucleocytoplasmic Large DNA Viruses (*s__Moumouvirus_moumou*) and crAss-like phages.

### Interactions Between Enteric Virome and Bacteriome in Patients With VKH 

Next, we analyzed the interactions between gut virome dysregulation and bacteriome dysbiosis in patients with VKH to determine their potential association. In order to facilitate subsequent correlation analyses, we categorized bacterial genera into core, common, and unique communities using the same criteria applied to viral communities,^27^ which defined the core bacterial community as those present in > 80% of samples, common in 50% to 80%, and unique in < 50%. Based on the classification criteria, the 59 differentially abundant genera identified through differential analysis were categorized into 9 core bacteria, 18 common bacteria, and 32 unique bacteria (Supplementary Fig. S3A). The richness of the core bacterial community was notably increased in patients with VKH (Supplementary Fig. S3B), which was contrary to the alterations in the core viral or phage communities. However, the richness of the other two bacterial communities remained comparable between the two groups (see Supplementary Fig. S3B). Likewise, the Shannon index of three bacterial communities was similar across both groups (Supplementary Fig. S3C). These results suggest that changes in the core bacterial and viral communities may interact with each other.

**Figure 4.**
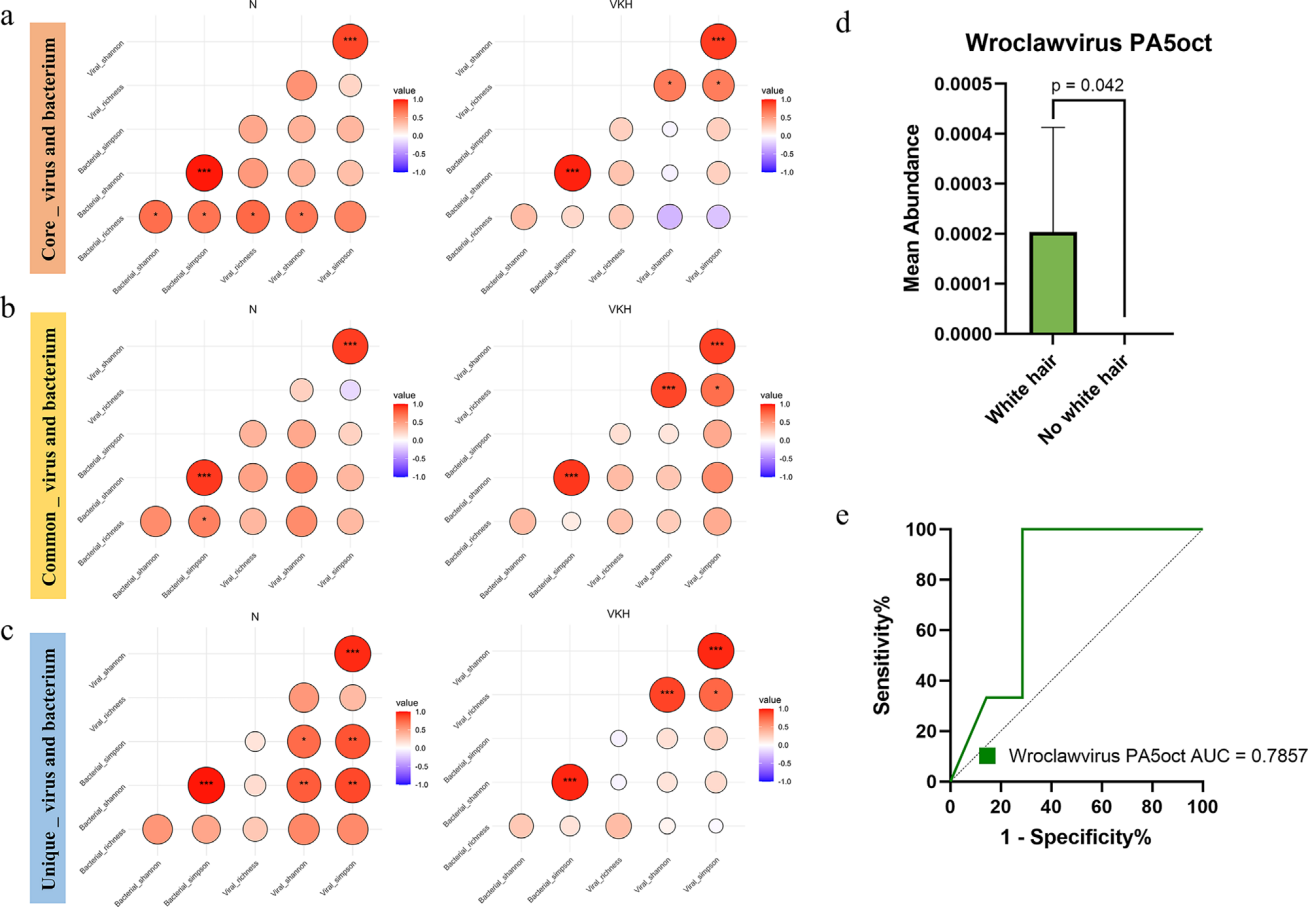
Enteric bacterial-viral correlation analysis in the VKH and the N groups. Correlations between the α diversity (richness, Shannon and Simpson) of core (**a**), common (**b**), and unique (**c**) viral and bacterial communities in healthy controls and UC, respectively. (**d**) Comparison of the relative abundance of Wroclawvirus PA5oct in groups with and without white hair. (**e**) Receiver operating characteristic (ROC) curve of Wroclawvirus PA5oct that distinguished the groups with and without white hair. *P < 0.05; **P < 0.01; ***P < 0.0001.

Correlation analysis of α diversity indices further revealed that the interactions between viruses and bacteria were weakened in patients with VKH compared with controls (Figs. 4a–c). Particularly in the core viral and bacterial communities, a positive correlation observed in the N group even shifted to a negative correlation in the VKH group (see Fig. 4a).

We also analyzed interactions between differentially abundant viruses and their bacterial hosts between the two groups. The results revealed that the interactions of *Colneyvirus CD27* (host: *Firmicutes*), *Seahorsevirus seahorse* (host: *Pseudomonadota*), and *Vhulanivirus Shpa* (host: *Pseudomonadota*) with their hosts were significantly weakened in the VKH group (see the Table).

**Table.** Correlation Analysis Between Differentially Abundant Viruses and Bacterial Hosts (Phylum)

Virus	Host_Bacteria (Phylum)	Correlation in VKH	Correlation in N
Kenoshavirus ohmyward	Actinobacteriota	0.19	0.20
Arthrobacter phage Phives	Actinobacteriota	−0.16	−0.30
Incheonvirus P12002L	Bacteroidota	0.57	0.25
Cellubavirus phi19una	Bacteroidota	0.36	0.17
Wulfhauvirus bangladeshii	Bacteroidota	0.09	0.48
Streptococcus phage PH15	Firmicutes	0.13	−0.16
Streptococcus phage PH10	Firmicutes	0.21	−0.59
Coventryvirus SpT152	Firmicutes	−0.07	−0.10
Vegasvirus vegas	Firmicutes	−0.48	−0.12
Unaquatrovirus uv1A4	Firmicutes	−0.03	−0.18
Colneyvirus CD27	Firmicutes	0.10	−0.66^*^
Siminovitchvirus JL	Firmicutes	−0.20	−0.42
Agatevirus agate	Firmicutes	−0.26	−0.18
Vibrio phage X29	Pseudomonadota	−0.13	−0.20
Seahorsevirus seahorse	Pseudomonadota	−0.18	**0.75** ^*^
Thalassomonas phage BA3	Pseudomonadota	0.24	0.26
Rhizobium phage 16-3	Pseudomonadota	0.25	0.50
Wroclawvirus PA5oct	Pseudomonadota	0.52	0.47
Catalunyavirus C5a	Pseudomonadota	−0.30	−0.13
Vhulanivirus Shpa	Pseudomonadota	−0.37	**0.93** ^***^
Mannheimia phage vB_MhS_535AP2	Pseudomonadota	0.19	0.21
Klebsiella virus KpV2811	Pseudomonadota	−0.32	0.21
Certrevirus CR3	Pseudomonadota	0.11	−0.02
Rauchvirus BPP1	Pseudomonadota	0.48	0.75
Chrysonvirus as5	Pseudomonadota	0.11	0.53
Obolenskvirus AbP2	Pseudomonadota	0.28	0.11

* *P* < 0.05.

*** *P* < 0.001.

The bolded value was used to highlight statistical significance.

Because *Pseudomonas* may be associated with depigmentation,^28^ and patients with VKH often present with symptoms of white hair, we next investigated the potential association between white hair and *Pseudomonas* bacteriophages. There are 16 bacteriophages that target *Pseudomonas*, with only *Wroclawvirus PA5oct* significantly reduced in the VKH group. The patients with VKH were divided into the white hair group (*n* = 7) and the non-white hair group (*n* = 3). There was no significant difference in age (*P* = 0.45) and gender (χ² = 1.270, *P* = 0.26) between the two groups. The relative abundance of *Wroclawvirus PA5oct* was significantly increased in the white hair group (*P* = 0.042; Fig. 4d). The AUC value of *PA5oct* could distinguish white hair from non-white hair patients (Fig. 4e).

### Functions of Enteric Virome in Patients With VKH

**Figure 5.**
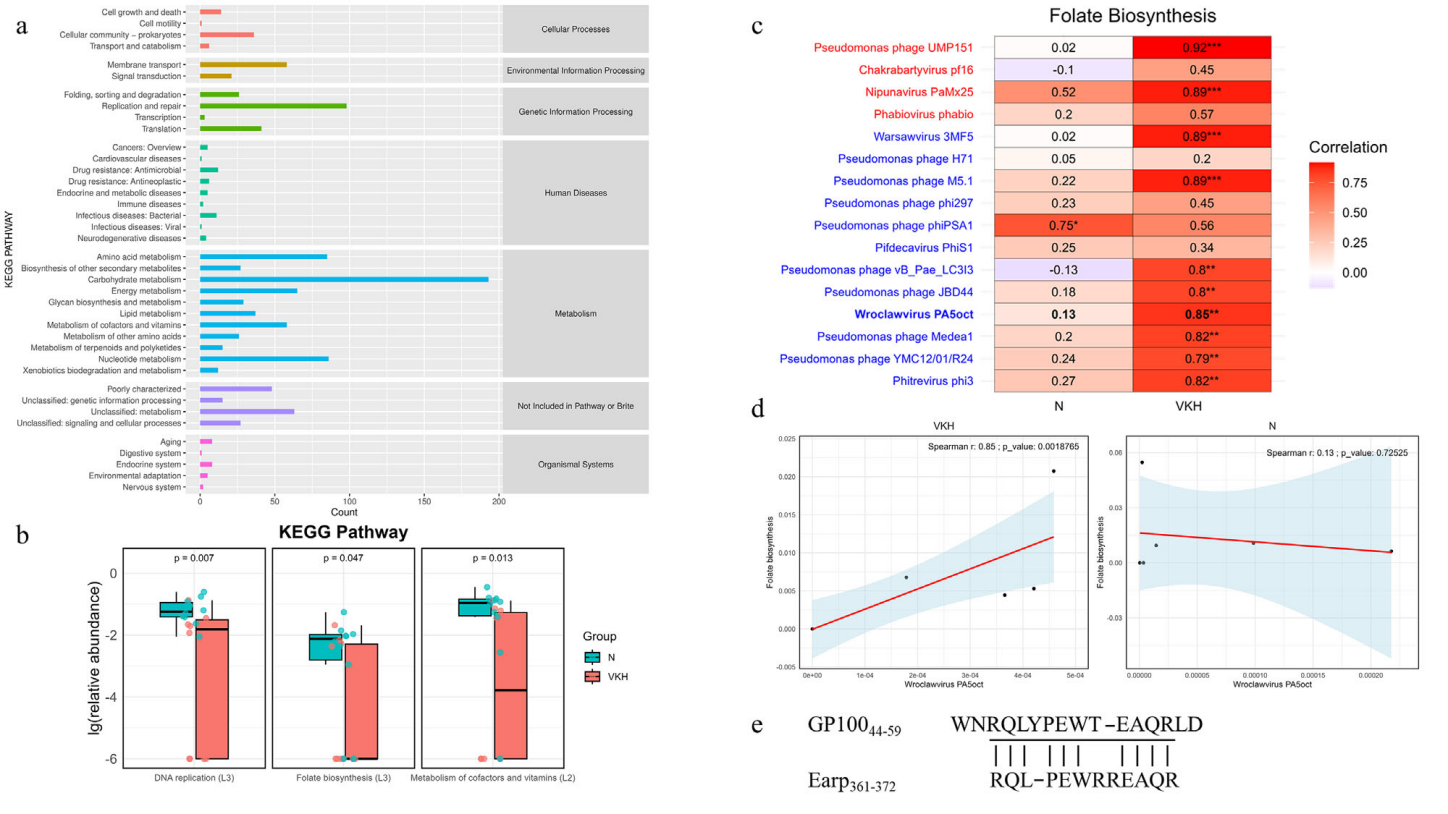
Differential virome functions between the VKH and the N groups. (**a**) Distribution of protein sequences annotated to the seven KEGG pathways. (**b**) The differential virome functions between the VKH and the N groups identified via the Wilcoxon test (*P* < 0.05). (**c**) Spearman correlation between the pathway of folate biosynthesis and the abundance of Pseudomonas phages. Phages color-coated in *blue* denote a relative reduction in the VKH group, whereas those color-coated in *red* denote a relative increase in the VKH group. (**d**) Spearman correlation between the pathway of folate biosynthesis and the abundance of Wroclawvirus PA5oct. (**e**) Schematic diagram of the protein Earp_361-372_ homology with gp100_44-59_.

KEGG annotation analysis using KOBAS was used to evaluate the potential functions of the gut virome in patients with VKH. A total of 755 (48.87%) protein sequences were annotated to 11 metabolic systems (Fig. 5a). Two core KEGG pathways (DNA replication and Cysteine and methionine metabolism), 33 common pathways, and 38 unique pathways were identified in the gut virome. Differential pathway analysis revealed that the metabolism of cofactors and vitamins (level 2), DNA replication (level 3), and Folate biosynthesis (level 3) were significantly downregulated in the VKH group (Fig. 5b).

Subgroup analysis indicated that the relative abundance of *PA5oct* differed significantly between patients with VKH with and without white hair. We proceeded to explore whether *Pseudomonas* phages are involved in mediating downregulated functional pathways in patients with VKH. Compared to the N group, the correlation between *Pseudomonas* bacteriophages and folate biosynthesis was markedly stronger in the VKH group (Fig. 5c). The results suggest that *PA5oct* has a notable impact on folate biosynthesis in the VKH group, whereas this effect is not observed in the N group (Fig. 5d).

To investigate potential cross-reactive antigens in the gut microbiota that may be involved in the pathogenesis of VKH, we analyzed the amino acid sequences homologous to the major antigenic epitopes of tyrosinase_450–462_ and gp100_44-59_ proteins. It was found that the glycosyltransferase A chain encoded by *Pseudomonas aeruginosa* (Earp_361-372_, RQLPEWRREAQR) showed high homology with gp100_44-59_ (WNRQLYPEWTEAQRLD; identity = 77%, E value = 3e-08; Fig. 5e).

## Discussion

In this study, we found that in patients with VKH, the core viral community was reduced, while the common viral community expanded. Significant differences were observed in the gut virome composition, including a decrease in crAss-like phages and the eukaryotic virus *Moumouvirus_moumou*, as well as an enrichment of the *Chlamydiamicrovirus_CPG1* phage. Gut virus-bacteria correlation analysis revealed altered cross-domain interactions in patients with VKH, particularly between the two core microbial communities. Furthermore, these differential viruses exhibited a loss of various viral functions. Importantly, disordered *Pseudomonas* phages may contribute to depigmentation by affecting host folate biosynthesis in patients with VKH. Meanwhile, the Earp protein in *Pseudomonas* may contribute to VKH pathogenesis by potentially triggering immune responses against melanocyte antigens via molecular mimicry mechanisms.

The healthy gut virome, composed of core and common viral communities, is essential for maintaining the configuration and function of the gut microbiome.^29^ In this study, the overall configuration of the gut virome of patients with VKH was not significantly different from that of healthy controls. However, the richness of core and common viral communities was notably altered, suggesting that these viruses are vital for human health. Moreover, the core phage community richness was decreased in patients with VKH, whereas the core eukaryotic virus community richness was increased. This is similar to the results in ulcerative colitis (UC),^29^^,^^30^ suggesting that phages and eukaryotic viruses affect human health in distinct ways. Temperate phage amplification and virulent phage reduction have been observed in patients with IBD, revealing that phages shape bacterial composition through predator-symbiosis mechanisms.^31^ On the other hand, eukaryotic viruses may interact with other microbiome components or human cells.^30^ Previous studies on eukaryotic viruses have found that the pathogenesis of VKH may be associated with high loads of EBV, and CMV envelope glycoprotein H (which shares sequence homology with melanin antigens).^8^^–^^10^ However, this study did not find significant changes in the *Herpesviridae* family in patients with VKH. This may be due to the small sample size and the limited enrichment of eukaryotic viruses. Further research is needed to explore the roles of phages and eukaryotic viruses in the pathogenesis of VKH.

The gut virome is predominantly composed of phages,^32^ and a majority of phages were observed to be reduced in the VKH patient cohort. CrAss-like phages were initially discovered through metagenomic analysis and are present in approximately 50% of the human gut virome, accounting for up to 90% of viral sequences.^33^ According to the host prediction, crAss-like phages mainly infect bacteria of Bacteriodetes.^34^ Multiple crAss-like phages were significantly deleted in patients with VKH, and similar alterations have been also observed in other autoimmune diseases, such as RA, SLE, and IBD.^35^^,^^36^ It can be concluded that there is an association between crAss-like phages and autoimmune diseases, but further studies are necessary to investigate the causal relationship between their reduction and host bacteria or disease states. *Chlamydiamicrovirus_CPG1* is a virulent phage that infects Chlamydia. Its capsid protein Vp1 can inhibit the growth of Chlamydia trachomatis and the production of pro-inflammatory cytokines IL-8 and IL-1.^37^ *CPG1* was significantly enriched in patients with VKH, but it remains unclear whether infection by this pathogen is a risk factor for VKH. The IL-23/IL-17 axis-mediated Th17 cell differentiation has been identified as a key mechanism in the pathogenesis of VKH disease.^38^ In our study, we observed that the disruption of microbial interactions in the VKH group might contribute to this immune dysregulation. Specifically, correlation analysis results showed that, compared to the N group, the interactions among *Colneyvirus CD27*, *Seahorsevirus seahorse*, or *Vhulanivirus Shpa* and their corresponding bacterial hosts (at the phylum level) were significantly weakened in the VKH group. *Seahorsevirus seahorse*, a bacteriophage infecting *Vibrio*, has been shown to promote the production of pro-inflammatory cytokines (IL-6 and TNF-α), thereby activating inflammation pathways in macrophages.^39^^,^^40^ *Clostridioides difficile* (targeted by *Colneyvirus CD27*) infection in collagen-induced arthritis (CIA) mice demonstrated that *C. difficile* infection reduced the incidence of arthritis, supporting the existence of a protective gut-joint axis. Spontaneous clearance of *C. difficile* further influenced the numbers and function of Foxp3+ cells in the mesenteric lymph nodes, which are essential for immune tolerance.^41^ The disruption of interactions in the VKH group may affect the regulatory role of bacteriophages on their host bacteria, leading to gut immune imbalance. Further investigation, including IL-17 and Th17/Treg analysis, is needed to better understand the mechanisms by which these microbial changes impact immune function and the pathogenesis of VKH.

*Pseudomonas* phages may contribute to depigmentation in patients with VKH by disrupting the host folate biosynthesis. After ampicillin gavage treatment, FH mice showed aggravated depigmentation, and gut dysbiosis primarily dominated by *Pseudomonas* might be one of the contributing factors.^28^ This suggests a potential link between *Pseudomonas* and depigmentation. Studies have found that *PA5oct* exhibits antibacterial potential against *Pseudomonas aeruginosa* isolated from different types of infections.^42^ On the one hand, *PA5oct* induces proinflammatory responses in monocytes and enhances innate immune clearance by targeting LPS and type IV pilus receptors.^42^ On the other hand, *PA5oct* can also affect the formation of bacterial biofilm and mediate cell lysis.^43^ In this study, we found that *PA5oct* was significantly decreased in patients with VKH. Furthermore, gut microbiome analysis also confirmed a significant enrichment of *Pseudomonas* in patients with VKH.^4^^,^^6^ Hence, PA5oct reduction may lead to a significant enrichment of *Pseudomonas* abundance, thereby affecting depigmentation.

Folate is an essential cofactor for the conversion of homocysteine (Hcy) to methionine. Its deficiency leads to increased Hcy, which inhibits the activity of tyrosinase in the skin and interferes with normal melanin synthesis.^44^ It has been reported that deleted folate levels are associated with depigmentation, and serum levels of folate are reduced in patients with vitiligo.^45^ Additionally, correlation analysis showed that *Pseudomonas* phage may have a stronger effect on host folate synthesis in patients with VKH. Interestingly, sequence alignment revealed that the Earp protein of *Pseudomonas aeruginosa* shares homologous sequences with the melanin antigen gp100. This raises the possibility of a cross-reaction, which warrants further investigation to explore its potential implications for VKH pathogenesis.

A major limitation of this study is the small sample size and the lack of remission-phase patients, which limits the analysis of gut virome changes between active and remission phases in VKH. Second, some novel viruses may have been unrecognized because of limitations in the reference database. Third, the shotgun metagenomic sequencing in this study excels at capturing actively infecting viruses and integrated prophages but has limited capacity for detecting free viral particles and RNA viruses.^36^ Combining VLP sequencing or improved library preparation techniques may enable a more comprehensive analysis of the gut virome.

In conclusion, our findings highlight the importance of the enteric virome in the pathogenesis of VKH. The disturbance of core viral community richness in patients with VKH is characterized by phage depletion and eukaryotic virus amplification, along with changes in specific viral species. Disrupted intestinal phages may reshape the intestinal bacterial community and contribute to the development of VKH by affecting host-related functions and microbial antigens. Focusing on alterations in different components of the gut microbiome can provide deeper insights into the role of microbial communities in the pathogenesis of VKH.

## Supplementary Material

Supplement 1
