# Supplementary material for: Alterations of the Enteric Virome in Vogt-Koyanagi-Harada Disease
Source: Invest Ophthalmol Vis Sci. 2025 Jun 4;66(6):15. doi: 10.1167/iovs.66.6.15 (PMC12147045; doi:10.1167/iovs.66.6.15)
Supplement: Supplement 1 [file iovs-66-6-15_s001.pdf]

A

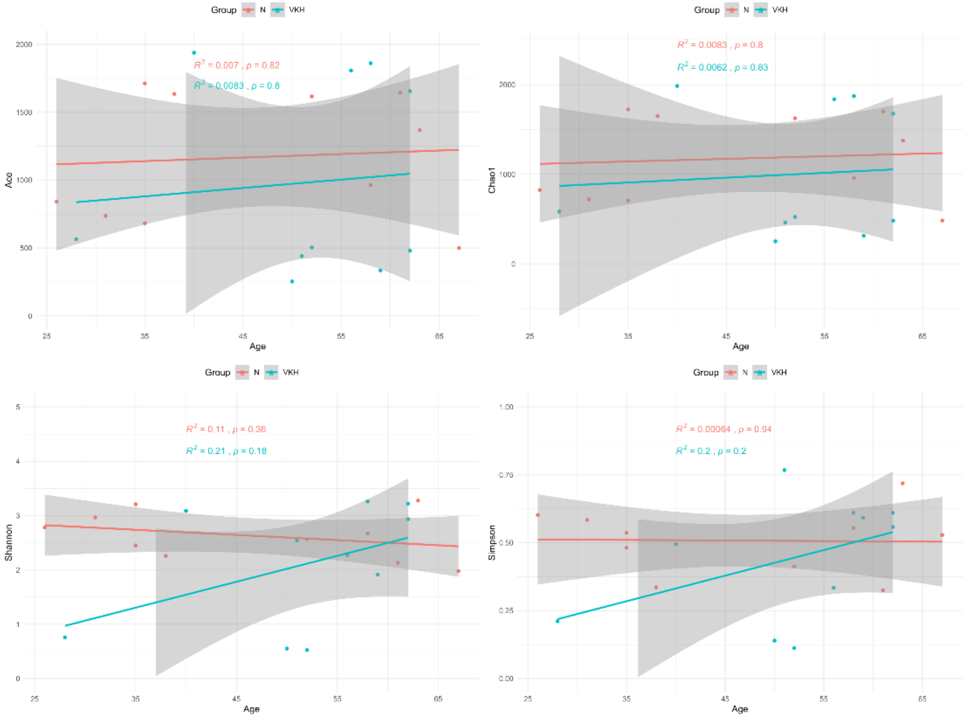

B

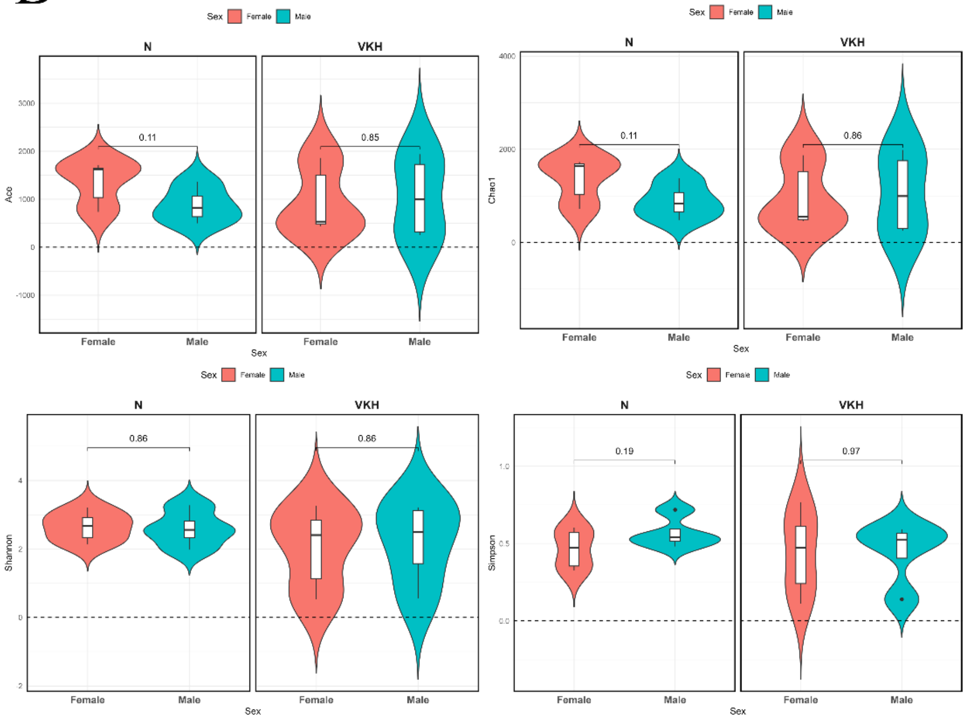

Supplementary Figure 1. The association of age and gender with gut viral alpha diversity in the VKH and N groups.

(A) Ace, Chao1, Shannon and Simpson indices correlate with age. Statistical significance was determined by linear regression. (B) Comparison of the four diversity indices between males and females.

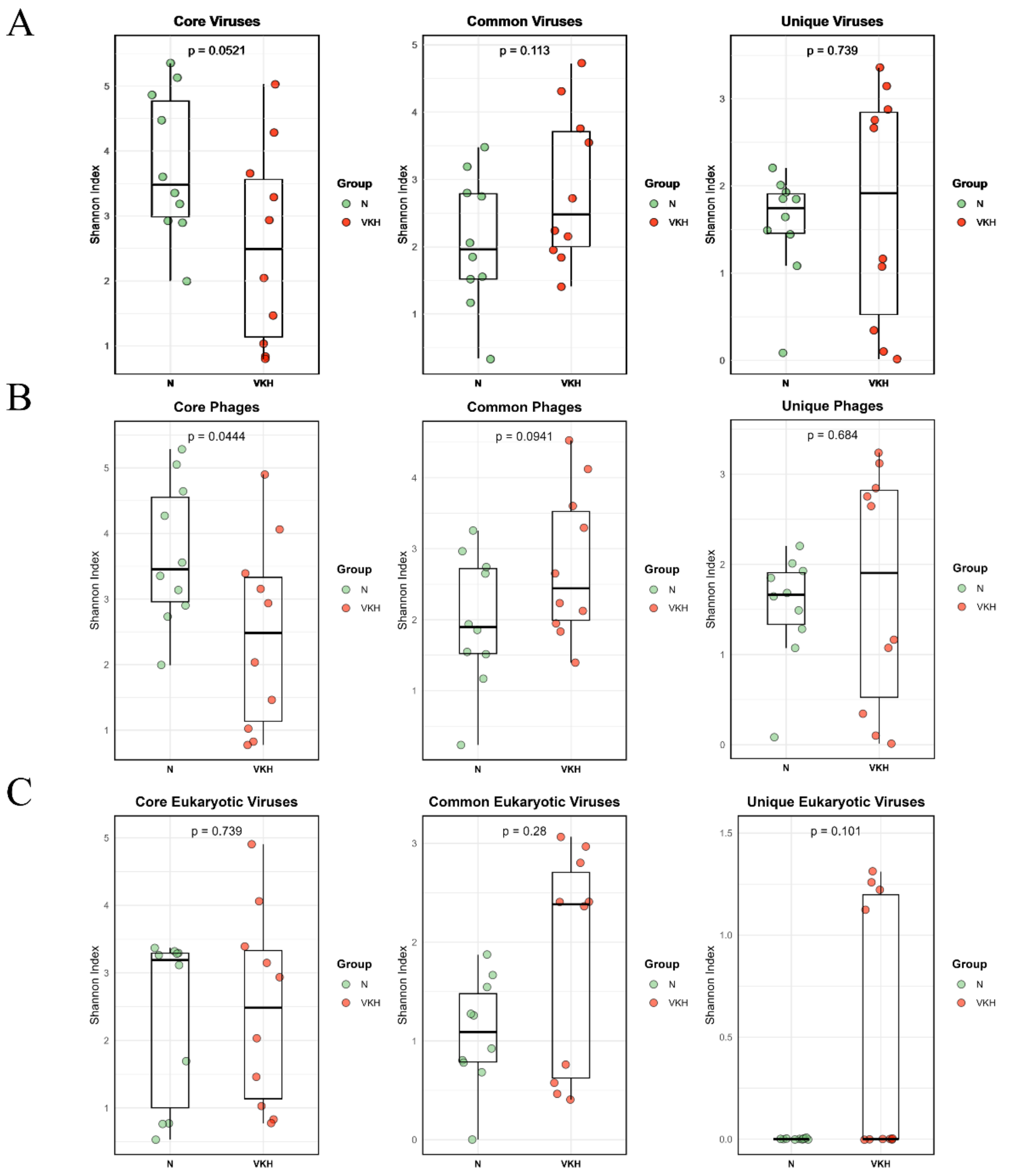

Supplementary Figure 2. The difference of the Shannon Index in the VKH and N groups.

(A) Shannon index analysis of three virus communities (core/common/unique) between the N and VKH groups. (B) Shannon index analysis of three phage communities (core/common/unique) between the N and VKH groups. (C) Shannon index analysis of three eukaryotic virus communities (core/common/unique) between the N and VKH groups.

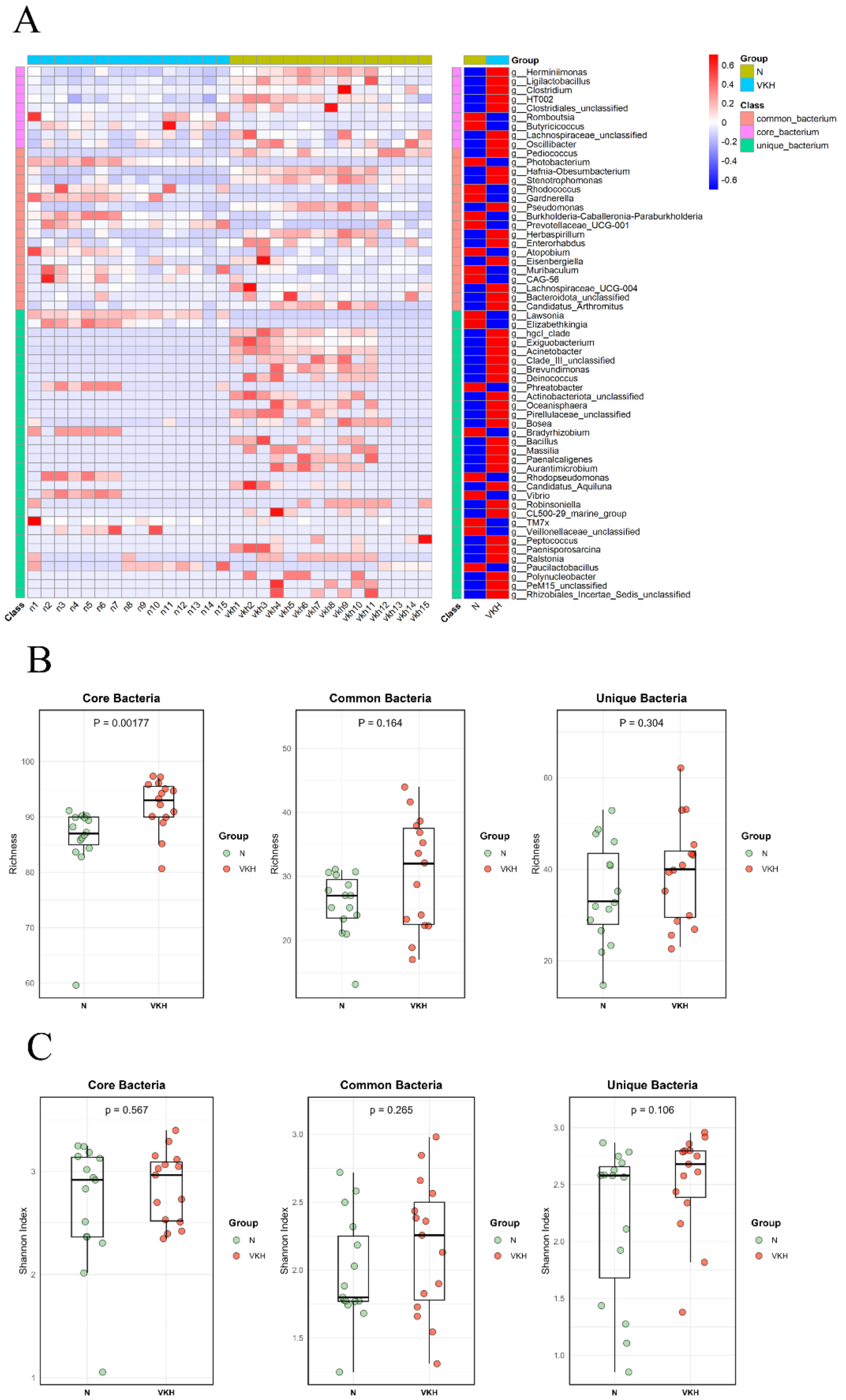

Supplementary Figure 3. The difference in bacterial community structure and taxonomic composition between the VKH and N groups.

(A) Differentially abundant bacteria at the genus level between VKH and N groups. (B) Richness analysis of three bacterial communities (core/common/unique) between the N and VKH groups. (C) Shannon index analysis of three bacterial communities (core/common/unique) between the N and VKH groups.
